# Applications of OptoProfilin in Living Cells for the Imaging of Focal Adhesions and Stress-Associated Phenotypes

**DOI:** 10.21769/BioProtoc.5841

**Published:** 2026-10-05

**Authors:** Clayton J. Brown, Robert M. Hughes

**Affiliations:** Department of Chemistry, East Carolina University, Greenville, NC, USA

**Keywords:** OptoProfilin, Profilin-1, Cry2, Cellular stress, Optogenetics, VASP, Focal adhesions, Biomolecular condensates

## Abstract

Cellular stress induces profound changes in cytoskeletal organization and biomolecular condensate formation. Traditional approaches for monitoring cellular stress often require multi-component biosensors, endpoint staining procedures, or indirect biochemical measurements. Here, we describe a protocol for the use of OptoProfilin, a genetically encoded single-component optogenetic biosensor derived from Profilin-1 fused to Cryptochrome 2 (Cry2) and mCherry. Following transient expression in mammalian cells, OptoProfilin exhibits light-dependent localization to focal adhesions under non-stressed conditions and transitions to punctate condensates under energetic, oxidative, osmotic, and senescence-associated stress conditions. The protocol includes transient transfection, induction of cellular stress, live-cell imaging, immunofluorescence validation, and quantitative image analysis. While this protocol describes imaging on a Leica widefield fluorescence microscope, it can readily be extended to other microscopy platforms. As a stand-alone biosensor that produces visually distinct responses in stressed versus non-stressed cells, OptoProfilin provides a convenient platform for investigating stress-associated cytoskeletal remodeling and biomolecular condensate formation.

Key features

• Single-component optogenetic biosensor for monitoring cellular stress.

• Distinguishes stressed and unstressed cells through distinct localization phenotypes.

• Compatible with live-cell imaging, fixed-cell analysis, and immunofluorescence.

• Applicable to energetic, oxidative, osmotic, and senescence-associated stress models.

## Graphical overview


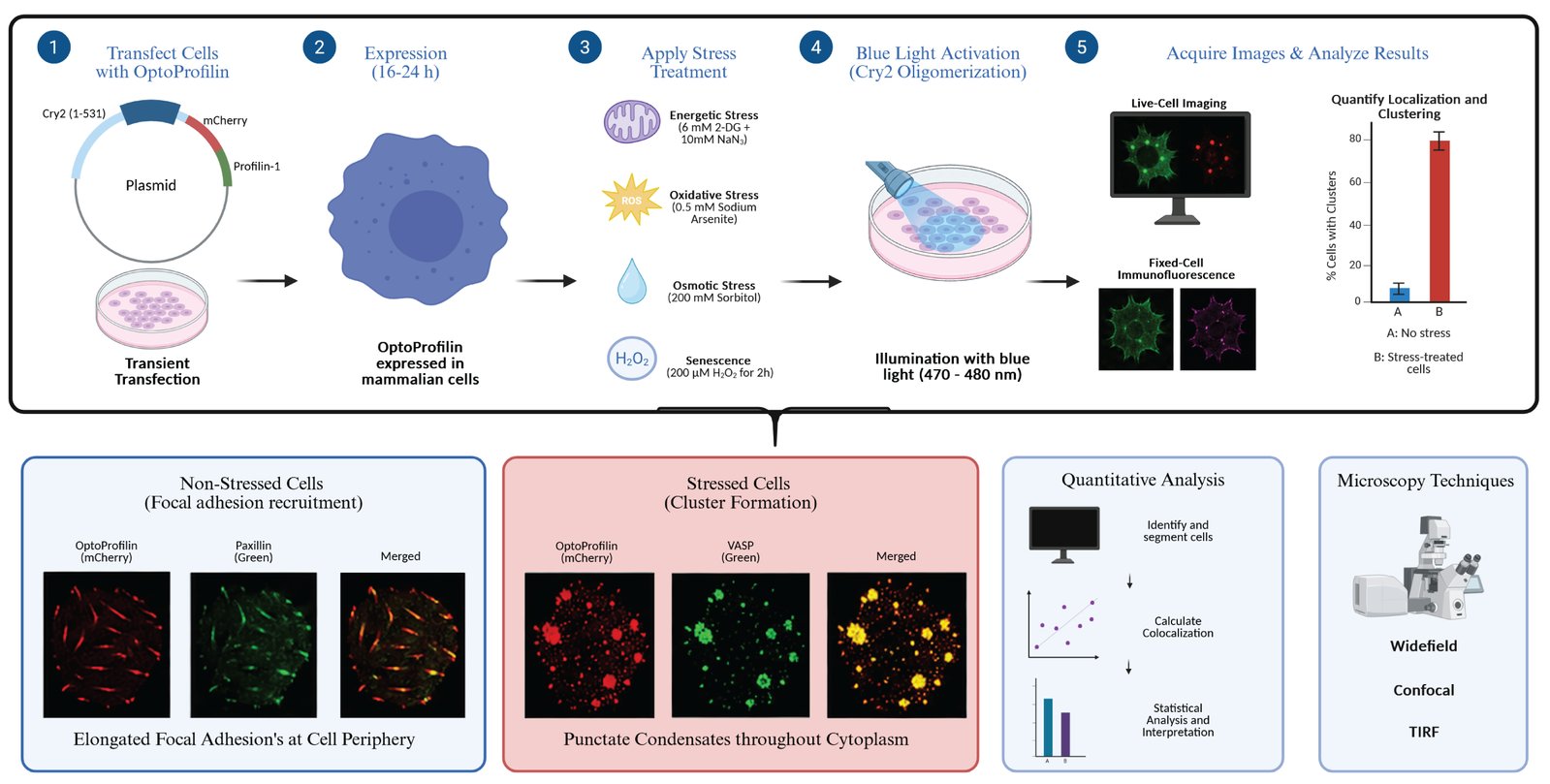


**OptoProfilin applications in cells.** Figure created using BioRender.

## Background

The actin cytoskeleton is highly responsive to environmental and physiological stress. Stress-induced remodeling of actin-associated proteins contributes to processes including biomolecular condensate formation, stress granule assembly, senescence, neurodegeneration, and disease progression. Profilin-1 is a multifunctional actin-binding protein that regulates actin polymerization and interacts with numerous cytoskeletal regulatory proteins, including vasodilator-stimulated phosphoprotein (VASP).

OptoProfilin was developed by fusing mouse Profilin-1 to the photolyase homology region of *Arabidopsis thaliana* Cryptochrome 2 and mCherry [1]. Upon blue-light activation, Cry2 undergoes oligomerization, promoting localization of OptoProfilin to VASP-containing structures; the interaction is reversible in the absence of blue light. Under non-stress conditions, OptoProfilin localizes primarily to focal adhesions. Following cellular stress, OptoProfilin transitions into punctate condensates that serve as a visual indicator of stress-associated cytoskeletal remodeling. OptoProfilin was first investigated using a Leica DMi8 live cell imaging system; thus, this system and the associated LasX software are featured prominently in this protocol. However, these methods could reasonably be extended to other microscopy platforms.

Compared to multi-component biosensors, OptoProfilin requires expression of only a single construct, simplifying experimental implementation and increasing compatibility with multiplexed imaging approaches. This protocol is best suited to energetic (ATP depletion), oxidative, osmotic, and senescence-associated stress paradigms; it is not appropriate for detecting heat stress, as OptoProfilin does not form clusters under heat shock conditions. VASP (vasodilator-stimulated phosphoprotein) is an actin-associated protein and a known component of focal adhesions. Mutations to the VASP-binding region of Profilin-1 within OptoProfilin eliminate both its focal adhesion and stress cluster phenotypes. In cell lines with higher VASP expression (e.g., N2a), the assay may lose its ability to discriminate stressed from unstressed cells. In cell lines with lower endogenous VASP expression (e.g., HEK293T, NIH 3T3), only the stress-induced clustering phenotype is informative, whereas the dual-phenotype readout (focal adhesion recruitment versus stress-induced clustering) is most robust in HeLa cells.

## Materials and reagents

**Biological materials**

1. OptoProfilin plasmids (Addgene plasmids, catalog numbers: 208286, 208287, 208288)

2. HeLa cells (ATCC, catalog number: CCL-2)

3. HEK293T cells (ATCC, catalog number: CRL-3216)

4. NIH 3T3 cells (ATCC, catalog number: CRL-1658)

5. Neuro-2a (N2a) cells (ATCC, catalog number: CCL-131)

6. Anti-VASP primary antibody (Cell Signaling, catalog number: 3132)

7. Anti-Paxillin primary antibody (Cell Signaling, catalog number: 12065)

8. Alexa Fluor 488-conjugated secondary antibody (Invitrogen, catalog number: A-11008)

**Reagents**

1. Dulbecco’s minimum essential medium (DMEM) with D-glucose and glutamine (Gibco, catalog number: 11965-092)

2. Fetalgro EX bovine growth serum (rmbio FGX-BBT) or fetal bovine serum (FBS) (Gibco, catalog number: A52568010)

3. Penicillin-streptomycin (Gibco, catalog number: 15140-122)

4. Trypsin-EDTA (0.25%), phenol red (Gibco, catalog number: 25200-056)

5. Calfectin transfection reagent (SignaGen, catalog number: SL100478)

6. Dulbecco’s phosphate-buffered saline (DPBS) containing Ca^2+^ and Mg^2+^ (Gibco, catalog number: 14040-133)

7. Sodium azide (NaN_3_) (Fisher Scientific, catalog number: BP9221)

8. 2-Deoxy-D-glucose (2-DG) (Sigma, catalog number: D8375)

9. Sodium (meta)arsenite (NaAsO_2_) (Sigma, catalog number: S7400)

10. Sorbitol (Fisher Scientific, catalog number: BP439)

11. Hydrogen peroxide (H_2_O_2_) (Sigma, catalog number: 216763)

12. Paraformaldehyde 16% (Fisher Scientific, Electron Microscopy Sciences, catalog number: 15710)

13. Triton X-100 (Acros, catalog number: 327372500)

14. Bovine serum albumin (BSA) (Fisher Scientific, catalog number: BP6711)

**Solutions**

1. ATP depletion medium (see Recipes)

2. Oxidative stress medium (see Recipes)

3. Osmotic stress medium (see Recipes)

4. Senescence induction medium (see Recipes)

5. Antibody dilution buffer (see Recipes)

6. 4% paraformaldehyde fixation solution (see Recipes)

**Recipes**

**1. ATP depletion medium**

**Table:** 

Reagent	Final concentration	Quantity or volume
DPBS (with Ca^2+^/Mg^2+^)	1×	9.84 mL
NaN_3_, 1 M stock	10 mM	100 μL
2-DG, 1 M stock	6 mM	60 μL
Total	n/a	10 mL

**2. Oxidative stress medium**

**Table:** 

Reagent	Final concentration	Quantity or volume
NaAsO_2_	0.5 mM	3.25 mg
DPBS	1×	to 50 mL
Total	n/a	50 mL

**3. Osmotic stress medium**

**Table:** 

Reagent	Final concentration	Quantity or volume
D-sorbitol	200 mM	1.82 g
DPBS	1×	to 50 mL
Total	n/a	50 mL

**4. Senescence induction medium**

**Table:** 

Reagent	Final concentration	Quantity or volume
H_2_O_2_, 9.79 M stock	200 μM	1.02 μL
DPBS	1×	49.999 mL
Total	n/a	50 mL

**5. Antibody dilution buffer**

**Table:** 

Reagent	Final concentration	Quantity or volume
1× PBS	1×	9.97 mL
BSA	1% (w/v)	0.1 g
Triton X-100	0.3% (v/v)	30 μL
Total	n/a	10 mL

**6. 4% paraformaldehyde fixation solution**

**Table:** 

Reagent	Final concentration	Quantity or volume
16% paraformaldehyde	4% (w/v)	10 mL
DPBS	0.75×	30 mL
Total	n/a	40 mL

**Laboratory supplies**

1. 35 mm glass-bottom dishes (MatTek, catalog number: P35G-1.5-14-C)

2. Sterile microcentrifuge tubes (1.5 mL)

3. Sterile serological pipettes

## Equipment

1. Tissue culture incubator (37 °C, 5% CO_2_)

2. Tissue culture microscope

3. Hemacytometer (Bright-Line^TM^ Hemacytometer; Cambridge Instruments, Inc.)

4. Widefield fluorescence microscope: Leica DMi8 Live Cell Imaging System, equipped with an OKOLab stage-top live cell incubation system, Infinity TIRF module, Leica HCX PL APO 63×/1.40–0.60 n.a. oil objective, Leica HC PL APO 100×/1.47 oil TIRF objective, Lumencor LED light engine, CTRadvanced+ power supply, and a Leica DFC900 GT camera

5. Confocal microscope (Zeiss LSM 700 laser scanning microscope using ZEN Black 2012 software)

6. Blue LED illumination source (Sunlite LED Par30 Reflector, Item #80021, 4 W, 120 V)

## Software and datasets

1. FIJI/ImageJ (ImageJ 1.54p 17 February 2025)

2. GraphPad Prism (10.6.1 (892) 31 October 2026)

3. ZEN Black 2012 or updated equivalent

4. LAS X software (3.7.6.25997)

## Procedure

**Part I. General live cell imaging of OptoProfilin using widefield microscopy**

**A. Preparation of HeLa cells for transfection (Figure 1)**

1. Grow HeLa cells in DMEM (10% FBS/1% Pen-Strep) at 37 °C and 5% humidity until they are 70%–80% confluent in a T75 growth flask with a filter cap.

2. In a laminar flow hood, remove growth media and wash cells 1× with 5 mL of DPBS.

3. Add 5 mL of trypsin reagent to promote cell detachment and return cells to the incubator. Use a microscope to check cells for detachment from the flask every 5 min.

4. When cells are fully detached, return to the flow hood and add 5 mL of growth media. Use a 10 mL serological pipette to fully dissociate cells by pipetting up and down several times. Remove a 1 mL aliquot of cells from the flask and place them in a sterile tube. Count these cells using a hemacytometer.

5. Plate 200,000 cells per glass-bottom Mattek dish, then add 2 mL of growth media via pipette. Put the cover on the dish and distribute the cells with a gentle rocking motion. Return to the incubator.

6. Allow cells to remain in the incubator overnight. The following day, check cells for confluency using a tissue culture microscope. Cells should be approximately 60%–70% confluent. We calculate this number by estimating the percentage of area in the field of view that is covered by adherent cells. At 70% confluency, most of the area will be covered with cells, with some gaps still remaining between individual cells or groups of adherent cells.

**Pause point:** If cells are not at the desired confluency, wait another 24 h before proceeding to the next step.

**Figure 1.**
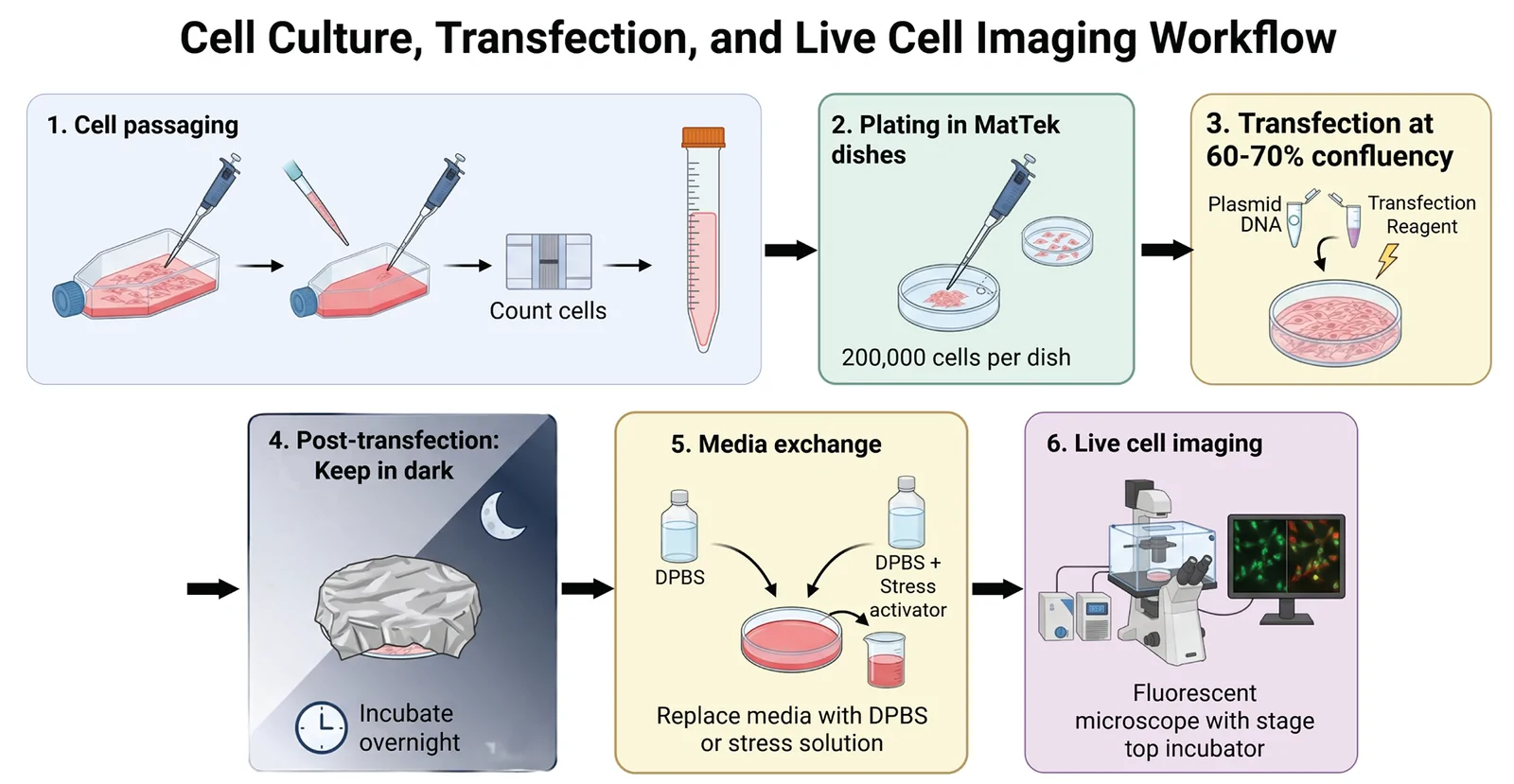
General workflow for OptoProfilin experiments. This workflow is described in greater detail in Part I. Figure created using BioRender (www.biorender.com).

**B. Transfection preparation (Figure 1)**

1. In a biological safety cabinet, aliquot 1,000 ng of OptoProfilin plasmid DNA into a sterile 1.5 mL tube.

2. Add 100 μL of serum-free DMEM to the DNA sample in the tube. Gently mix and spin down the solution with a benchtop centrifuge.

3. Add 3 μL of Calfectin transfection reagent to the DNA–DMEM solution. Gently mix by tapping the bottom of the tube and spin down the solution with a benchtop centrifuge.

**Critical:** Allow the transfection solution to sit for at least 10 but no longer than 20 min, per manufacturer’s recommendation, as longer incubation times lead to reduced transfection efficiency and higher cell toxicity.

4. Add the transfection solution to the cells in the Mattek dish dropwise, using a circular motion to disperse the drops evenly over the dish. Then, return the cover to the dish and rock gently back and forth. Wrap the dish with aluminum foil to prevent unintended light activation and return it to the incubator. The experiment can begin after 16 h of incubation with the transfection complexes.

*Note: This step can be scaled as needed for the required number of dishes/replicates.*

**C. Microscope preparation**

1. Turn on the microscope and the stage-top incubator. Open the LasX software on the workstation. The software will run through multiple checks, including the camera and the x, y, z stage. Make sure that the 10× objective is in imaging (upright) position prior to beginning these steps to protect the higher magnification objectives.

2. Place the stage-top incubator on the microscope. To minimize drift during the experiment, it is helpful to have the incubator and microscope assembly set up at least 1 h prior to imaging.

3. To prevent unintended light activation, it is helpful to have a red safe light for illumination. It is also possible to work under near-dark conditions if the imaging area is sequestered by a laser safety curtain and a dim light is present from an adjacent room.

4. Set the microscope to image in fluorescent mode with the DSRed-T channel (553 nm activation) with the 63× oil immersion objective.

**D. Cellular imaging**

**D1. Cell and microscope preparation for mounting and imaging (Figure 2**)

1. In a dark room with the red safe light on, remove cell culture media from the dish by pipette or aspirator.

2. Wash cells by carefully adding 500 μL of DPBS to the dish and remove by aspirator or pipette.

3. Carefully add 2 mL of DPBS to the dish and return the cover to the dish.

4. Add a drop of immersion oil to the 63× objective. Also, lightly coat the glass bottom of the Mattek dish with a layer of immersion oil.

5. Mount the dish over the 63× objective using the round sample holder portion of the stage-top incubator. The dish can be secured with magnetic clamps as needed.

6. Using the LasX software, turn on live imaging mode and locate the transfected cells using the DSRed channel (200 ms exposure time and 50% LED power), as shown in **Figure 2A**.

*Note: It is very helpful to have a “locator” dish of cells (can be live or fixed) to get the objective in the correct position prior to locating live fluorescent cells. Change the contrast to the “AutoScale” setting when locating cells, as this will make brighter cells stand out.*

**Figure 2.**
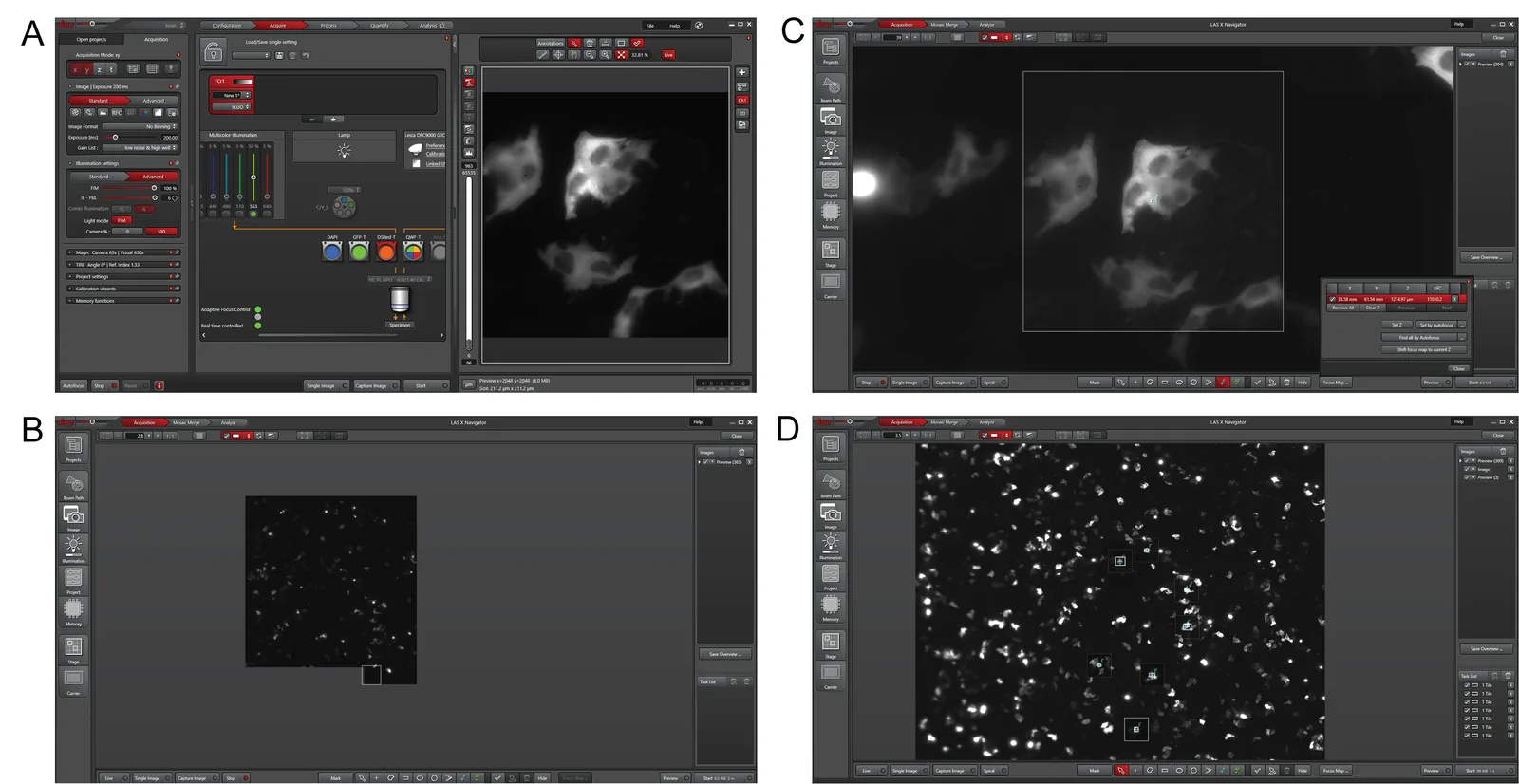
Identification of OptoProfilin expressing cells on the Leica DMi8 LAS X platform. (A) LAS X image acquisition interface. Image acquisition settings are on the left, optical channel settings in the middle, and image viewer on the right. In this panel, cells have been located with the DSRed-T fluorescent channel and 63× oil immersion objective. (B) View of *Spiral acquisition* mode in progress in the LAS X Navigator window. (C) Setting of focus map points and acquisition tasks in the LAS X Navigator. All manipulations are done using the DSRed-T channel to avoid preliminary activation of OptoProfilin. (D) The completed cell grid acquired with *Spiral Mode* with seven tasks and focus map points set. At this point, the timed experiment can be set up and executed.

7. Once red fluorescent cells have been located, set the z focal plane and turn off live imaging for 2–3 min to allow the cells to equilibrate in the stage-top incubator.

8. Turn live imaging back on, correct the focus, and turn live imaging back off for 2–3 min. Repeat this step as needed until focus has stabilized. Total time for this process is 10–15 min.

9. Turn on live imaging and go to the LAS *Navigator* panel (a gridded button on the *Acquisition Panel*). Using a 200 ms exposure time and the DSRed-T fluorescent channel (these settings will carry over if set up in previous steps), activate *Spiral mode*, as shown in **Figure 2B**. This will collect a grid of images that can be used to locate fluorescent cells quickly.

10. Once the spiral imaging has completed, double-click on the cells of interest in live mode, correct focus, set the focal plane (to cell groups of interest, add a *Focus Map Point*, go to the *Focus Map*, highlight the relevant map point, select *Set Z*), and use the rectangular selection tool to add the cell to the task list, as shown in **Figure 2C, D**. Do this for up to 10 cells or 10 regions of cells. Once cells are selected, with live mode on, click through all of the *Focus Map* points in the *Focus Map* to check that the cells have not gone out of focus. Adjust as necessary and select *Set Z* to update the focus setting.

**Critical:** At this point, it is important to turn live mode off. In the next steps, the GFP channel will be turned on (480 nm). This could inadvertently activate your cells if live mode is in the ON position.

11. Use the LasX software to add a second fluorescent channel (**Figure 3**). Set this channel to image GFP and turn the 480 nm LED to 50% power. Set the exposure time for this channel to 50 ms.

*Note: A longer exposure time is used for DSRed to capture the fainter mCherry, and a shorter exposure time is used for GFP since it is for light activation only. mCherry excitation might be a better choice if available, and the 50 ms GFP exposure could be further reduced if blue light-associated cytotoxicity is of concern.*

**Figure 3.**
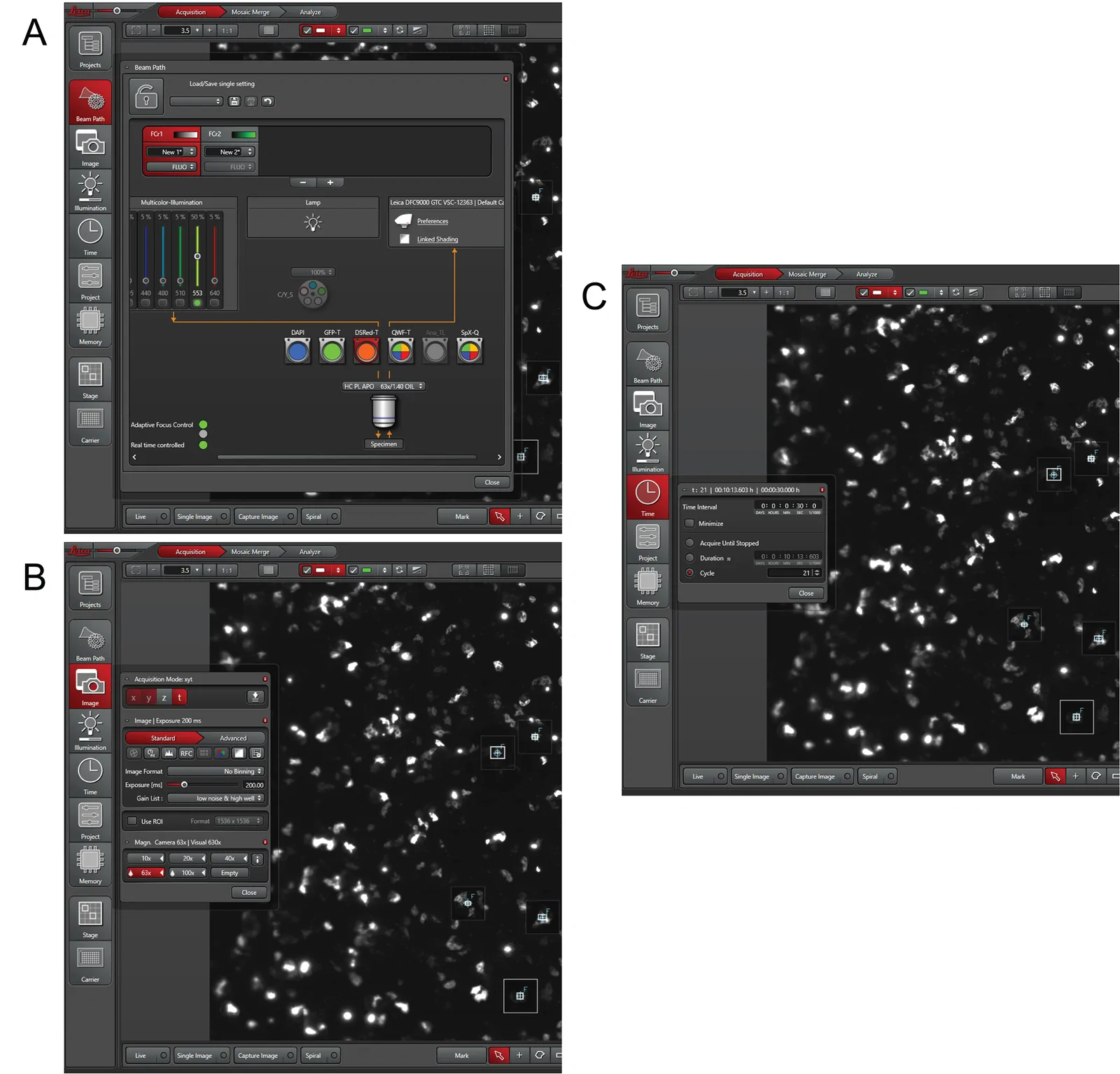
Setting up image capture and sequence parameters on the Leica DMi8 LAS X platform. (A) Adding the GFP channel in LAS Navigator. Note that live mode is off during these steps. (B) Setting the timed acquisition mode (“t” should be highlighted). (C) Setting the frequency and duration of imaging.

12. Set up the timed experiment by adding a time series in the *Acquisition* panel. Set the interval to 30 s and the number of frames acquired to 21. The total acquisition time for this experiment will be approximately 10 min.

13. The images will be collected in the order that the fluorescent channels are set up in the software. Make sure that the DSRed channel is first and the GFP channel is second. This gives one image of cells prior to blue light activation.

14. Click on *Start* to begin the experiment. Recruitment to focal adhesions will be visible by the second or third frame in the timed sequence.

**Critical:** Capturing the correct focal plane for focal adhesions in widefield imaging is non-trivial and may require some trial and error. For some cells, the focal plane will be correct the first time. For others, it will be helpful to activate the cell, refocus to find the focal adhesions, and then allow the cells to recover in the dark prior to beginning the imaging sequence again. This difficulty is largely eliminated in TIRF mode, which is described further below.

15. After the 10-min acquisition sequence is complete, quickly remove the GFP acquisition channel from the software, and begin the acquisition again. This will enable imaging of OptoProfilin as it returns from the focal adhesions back to the cytosol. Dark reversion of OptoProfilin from focal adhesions has a t_1/2_ of 150 (±10) s, and a 5-min waiting period is sufficient prior to performing another reactivation experiment if desired.

*Note: Many variations of this experiment can be performed (cycling blue light off and on several times; changing activation intervals and exposure times; changing LED power). These are not explicitly described here, but are useful for benchmarking light-responsive properties of OptoProfilin across various microscopes and cell types.*

**D2. General cellular stress testing of OptoProfilin using widefield microscopy**

1. In a dark room with the red safe light on, remove the cell culture media from the dish by pipette or aspirator.

2. Wash cells by carefully adding 500 μL of DPBS to the dish and remove by aspirator or pipette.

3. Carefully add 2 mL of cellular stress media to the dish and return the cover to the dish. This media could be the ATP depletion media (NaN_3_/2-DG), oxidative stress media (sodium arsenite), osmotic stress media (sorbitol), or the reagent of your choice. The stress cluster phenotype can be distinguished by its brighter and more punctate clusters versus the focal adhesion phenotype, which is fainter and more wedge-like (**Figure 4**).

**Figure 4.**
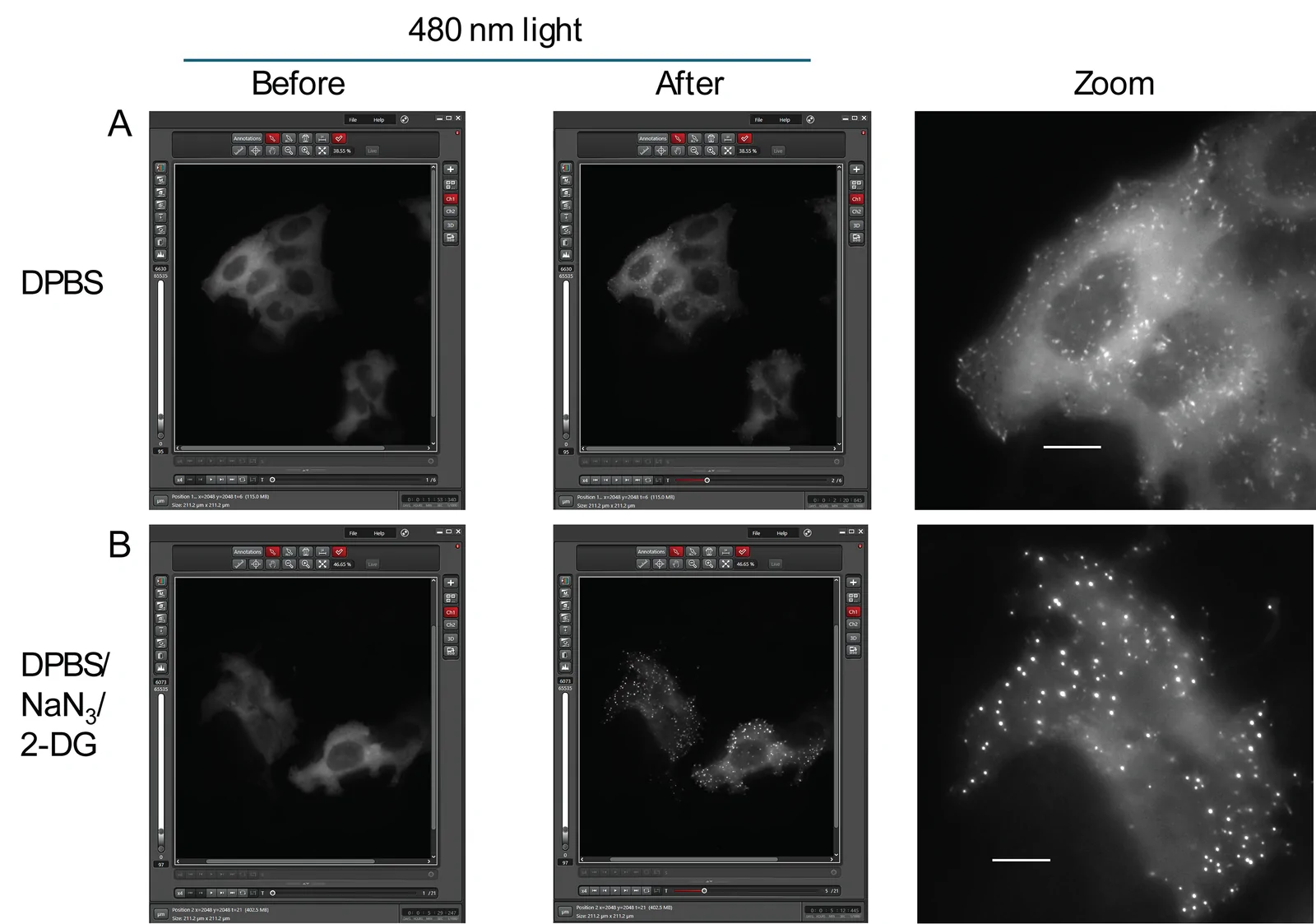
Comparison of non-stress and stress phenotypes of OptoProfilin. (A) Cells before and after 480-nm light exposure (left, center) and a zoomed image of a recruited cell (right). Scale bar = 10 μm. (B) ATP depletion media–treated cells before and after 480-nm light exposure (left, center) and a zoomed image of a recruited cell (right). Scale bar = 10 μm. For examples of the OptoProfilin response to other types of cell stress media, see [1].

4. Add a drop of immersion oil to the 63× objective. Lightly coat the glass bottom of the Mattek dish with a layer of immersion oil.

5. Mount the dish over the 63× objective using the round sample holder portion of the stage-top incubator. The dish can be secured with magnetic clamps as needed.

6. Using the LasX software, turn on live imaging mode and locate the transfected cells using the DSRed channel (200 ms exposure time and 50% LED power).

*Note: It is very helpful to have a “locator” dish of cells (can be live or fixed) to get the objective in the correct position prior to locating live fluorescent cells. Change the contrast to the “auto” setting when locating cells, as this will make brighter cells stand out.*

7. Once red fluorescent cells have been located, set the z focal plane and turn off live imaging for 2–3 min to allow the cells to equilibrate in the stage-top incubator.

8. Turn live imaging back on, correct the focus, and turn live imaging back off for 2–3 min. Repeat this step as needed until focus has stabilized. Total time for this process is 10–15 min.

9. Turn on live imaging and go to the LAS Navigator panel. Using a 200-ms exposure time, activate *Spiral mode*. This will collect a grid of images that can be used to locate fluorescent cells quickly.

10. Once the spiral imaging has completed, double-click on cells of interest in live mode, correct focus, set the focal plane, and use the rectangular selection tool to add the cell to the task list. Do this for up to 10 cells or 10 regions of contiguous cells.

11. Follow the remaining steps as described in part A for image acquisition.

12. After the 10-min acquisition sequence is complete, quickly remove the GFP acquisition channel from the software, and begin the acquisition again. This will enable imaging of OptoProfilin as it returns from the stress clusters back to the cytosol. Dark reversion of OptoProfilin from stress clusters has a t_1/2_ of 190 (±10) s, and a 6-min waiting period is sufficient prior to performing another reactivation experiment if desired.

*Note: Cells should incubate in stress media for a minimum of 10 min; this incubation period includes the time required to establish focus and cell positions with the imaging system.*

**D3. Mid-sequence application of cellular stress to OptoProfilin using widefield microscopy (Figure 5)**

1. In a dark room with the red safe light on, remove the cell culture media from the dish by pipette or aspirator.

2. Wash cells by carefully adding 500 μL of DPBS to the dish and remove it with an aspirator or pipette.

3. Carefully add 2 mL of DPBS to the dish; do not return the cover to the dish.

4. Add a drop of immersion oil to the 63× objective. Also, lightly coat the glass bottom of the Mattek dish with a layer of immersion oil.

5. Mount the *uncovered* dish over the 63× objective using the round sample holder portion of the stage-top incubator. The dish can be secured with magnetic clamps as needed.

6. Using the LasX software, turn on live imaging mode and locate the transfected cells using the DSRed channel (200 ms exposure time and 50% LED power).

*Note: It is very helpful to have a “locator” dish of cells (can be live or fixed) to get the objective in the correct position prior to locating live fluorescent cells. Change the contrast to the “auto” setting when locating cells, as this will make brighter cells stand out.*

7. Once red fluorescent cells have been located, set the z focal plane and turn off live imaging for 2–3 min to allow the cells to equilibrate in the stage-top incubator.

8. Turn live imaging back on, correct the focus, and turn live imaging back off for 2–3 min. Repeat this step as needed until focus has stabilized. Total time for this process is 10–15 min.

9. Turn on live imaging and go to the LAS Navigator panel. Using a 200-ms exposure time, activate *Spiral mode*. This will collect a grid of images that can be used to locate fluorescent cells quickly.

10. Once the spiral imaging has completed, double-click on cells of interest in live mode, correct focus, set the focal plane, and use the rectangular selection tool to add the cell to the task list. Do this for up to 10 cells or 10 regions of contiguous cells.

**Critical:** At this point, it is important to turn live mode off. In the next steps, the GFP channel will be turned on (480 nm). This could inadvertently activate your cells if live mode is in the ON position.

11. Use the LasX software to add a second fluorescent channel. Set this channel to image GFP and turn the 480 nm LED to 50% power. Set the exposure time for this channel to 50 ms.

12. Set up the timed experiment by adding a time series in the *Acquisition* panel. Set the interval to 30 s and the number of frames acquired to 11. These first frames will provide a baseline for the cellular response in the absence of cellular stress.

13. The images will be collected in the order that the fluorescent channels are set up in the software. Make sure that the DSRed channel is first and the GFP channel is second. This gives one image of cells prior to blue light activation.

14. Click on *Start* to begin the experiment. Recruitment to focal adhesions will be visible by the second or third image acquisition in the timed sequence.

15. At the completion of the 5-min interval, carefully open the laser containment shell and gently push back the top of the microscope. Remove the cover from the stage-top incubator and set it aside. Without disturbing the dish, carefully pipette 200 μL of a 10× stock of cellular stress media onto the cells (for example, 100 mM NaN3/60 mM 2-DG).

**Critical:** It is important to do so carefully so that the cellular positions do not change; some fluctuation in z is to be expected, but shifts in x and y positions will be detrimental to the experiment. After the media has been added, lower the microscope head and close the laser protection cover. Check the focus on the DSRed channel and adjust as needed.

16. Set up the second portion of the timed experiment by adding a time series in the *Acquisition* panel. Set the interval to 30 s and the number of frames acquired to 21. These frames will reveal post-stress changes in OptoProfilin recruitment. As quickly as possible, begin the acquisition.

**Figure 5.**
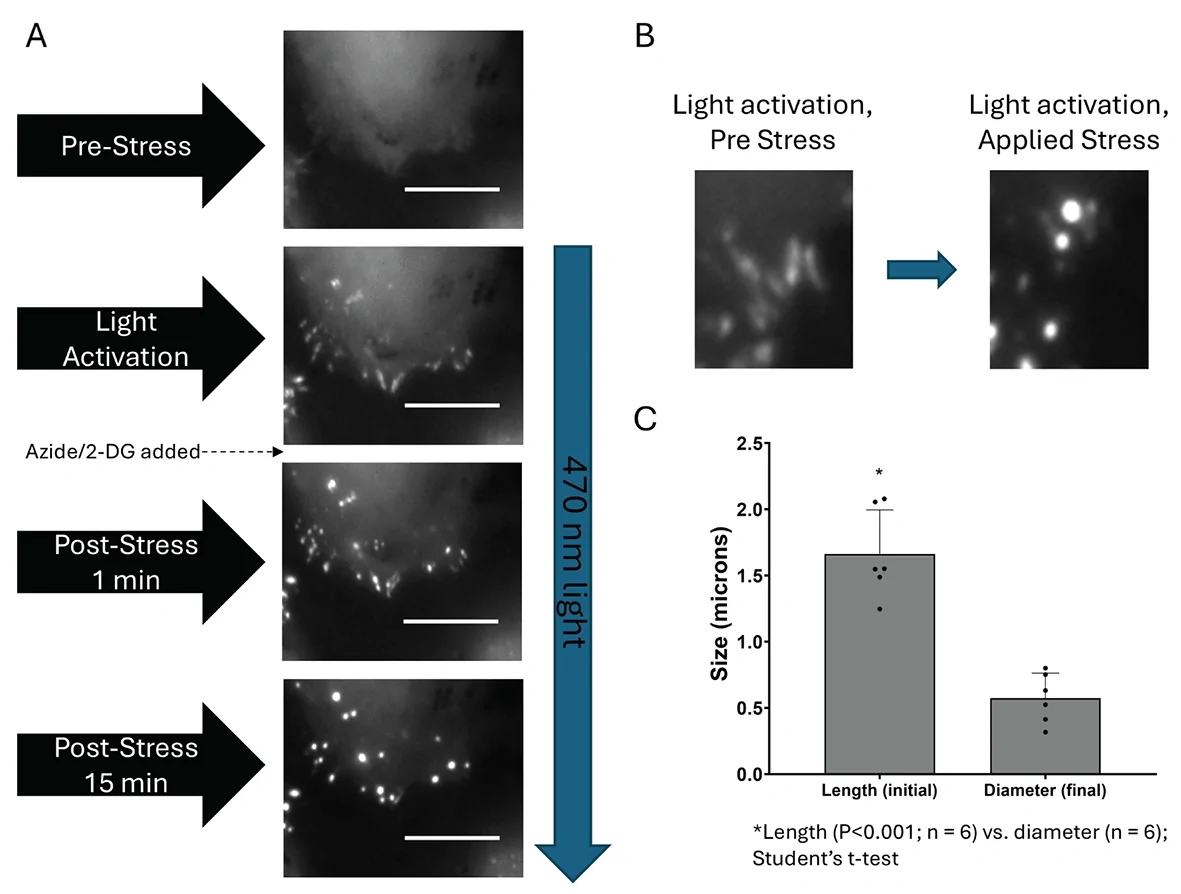
Stress-activated, vasodilator-stimulated phosphoprotein (VASP)-mediated focal adhesion to cluster transition. (A) OptoProfilin recruitment to elongated focal adhesion followed by addition of ATP-depletion media (final concentration 10 mM NaN3/6 mM 2-DG) and imaging of subsequent transition to VASP-rich clusters in HeLa cells. (B) Close-up of transition to stress-induced phenotype. (C) Quantification of the stress-induced focal adhesion transition observed in panel B. Scale bars = 10 μm. Figure adapted from [1].

**Part II. General live cell imaging of OptoProfilin using TIRF microscopy**

**A. Preparation of HeLa cells for transfection**

1. Grow HeLa cells in DMEM (10% FBS/1% Pen-Strep) at 37 °C and 5% humidity until they are 70%–80% confluent in a T75 growth flask with filter cap.

2. In a laminar flow hood, remove growth media and wash cells 1× with 5 mL of DPBS.

3. Add 5 mL of trypsin to promote cell detachment and return cells to the incubator. Use a microscope to check cells for detachment from the flask every 5 min.

4. When cells are fully detached, return to the flow hood and add 5 mL of growth media. Use a 10 mL serological pipette to fully dissociate cells by pipetting up and down several times. Remove a 1 mL aliquot of cells from the flask and place them in a sterile tube. Count these cells using a hemacytometer.

5. Plate 200,000 cells per glass-bottom Mattek dish, then add 2 mL of growth media via pipette. Put the cover on the dish and distribute cells with a gentle rocking motion. Return to the incubator.

6. Allow cells to remain in the incubator overnight. The following day, check cells for confluency using a tissue culture microscope. Cells should be approximately 60%–70% confluent.

**Pause point:** If cells are not at the desired confluency, wait another 24 h before proceeding to the next step.

**B. Transfection preparation**

1. In a biological safety cabinet, aliquot 1,000 ng of OptoProfilin plasmid DNA into a sterile 1.5 mL tube.

2. Add 100 μL of serum-free DMEM to the DNA sample in the tube. Gently mix and spin down the solution with a benchtop centrifuge.

3. Add 3 μL of Calfectin transfection reagent to the DNA–DMEM solution. Gently mix by tapping the bottom of the tube and spin down the solution with a benchtop centrifuge.

**Critical:** Allow transfection solution to sit for at least 10 but no longer than 20 min, per manufacturer’s recommendation, as longer incubation times lead to reduced transfection efficiency and higher cell toxicity.

4. Add the transfection solution to the cells in the Mattek dish dropwise, using a circular motion to disperse the drops evenly over the dish, then return the cover to the dish and rock gently back and forth. Wrap the dish with aluminum foil to prevent unintended light activation and return it to the incubator. The experiment can begin after 16 h of incubation with the transfection complexes.

*Note: This step can be scaled as needed for the required number of dishes/replicates.*

**C. Microscope preparation**

1. Prior to turning on the microscope, turn on the UV laser and turn the key to the ON position.

2. Turn on the microscope and the stage-top incubator. Open the LasX software on the workstation. The software will run through multiple checks, including the camera and the x, y, z stage. Make sure that the 10× objective is in imaging (upright) position prior to beginning these steps to protect the higher magnification objectives.

3. Place the stage-top incubator on the microscope. To minimize drift during the experiment, it is helpful to have the incubator and microscope assembly set up at least 1 h prior to imaging.

4. To prevent unintended light activation, it is helpful to have a red safe light for illumination. It is also possible to work under near dark conditions if the imaging area is sequestered by a laser safety curtain and a dim light is present from an adjacent room.

5. Set the microscope to image in fluorescent mode with the DSRed-T channel (553 nm activation) with the 100× oil immersion objective.

*Note: 100×/1.47 is the high numerical aperture objective on this system dedicated to TIRF. Other platforms may use high NA objectives (NA > 1.45) for TIRF.*

**D. Cell and microscope preparation for mounting and imaging**

1. In a dark room with the red safe light on, remove the cell culture media from the dish by pipette or aspirator.

2. Wash cells by carefully adding 500 μL of DPBS to the dish and remove by aspirator or pipette.

3. Carefully add 2 mL of DPBS to the dish and return the cover to the dish.

4. Add a drop of immersion oil to the 100× objective. Also, lightly coat the bottom of the Mattek dish with a layer of immersion oil.

5. Mount the dish over the 100× objective using the round sample holder portion of the stage-top incubator. The dish can be secured with magnetic clamps as needed.

6. Using the LasX software, turn on live imaging mode and locate the transfected cells using the DSRed-T fluorescent channel (200 ms exposure time and 50% LED power).

*Note: It is very helpful to have a “locator” dish of cells (can be live or fixed) to get the objective in the correct position prior to locating live fluorescent cells. Change the contrast to the “auto” setting when locating cells, as this will make brighter cells stand out.*

7. Once red fluorescent cells have been located, set the z focal plane and turn off live imaging for 2–3 min to allow the cells to equilibrate in the stage-top incubator.

8. Turn live imaging back on, correct the focus, and turn live imaging back off for 2–3 min. Repeat this step as needed until focus has stabilized. Total time for this process is 10–15 min.

9. Set the red fluorescent channel to TIRF mode (select widefield laser to 561 nm at 10% power with the DSRed-T filter cube; put TIRF in EPI mode to begin). Turn live imaging on, confirm that fluorescence is visible in the EPI mode, and then set the TIRF penetration depth and angle using the TIRF settings window.

*Note: The optimal TIRF angle is the one that provides the brightest but least EPI-like image of the cell while minimizing the appearance of alternating areas of high and low fluorescence intensity and the appearance of concentric rings (****Figure 6***).

**Critical:** At this point, it is important to turn live mode off. In the next steps, the GFP channel will be turned on (480 nm). This could inadvertently activate your cells if live mode is in the ON position.

**Figure 6.**
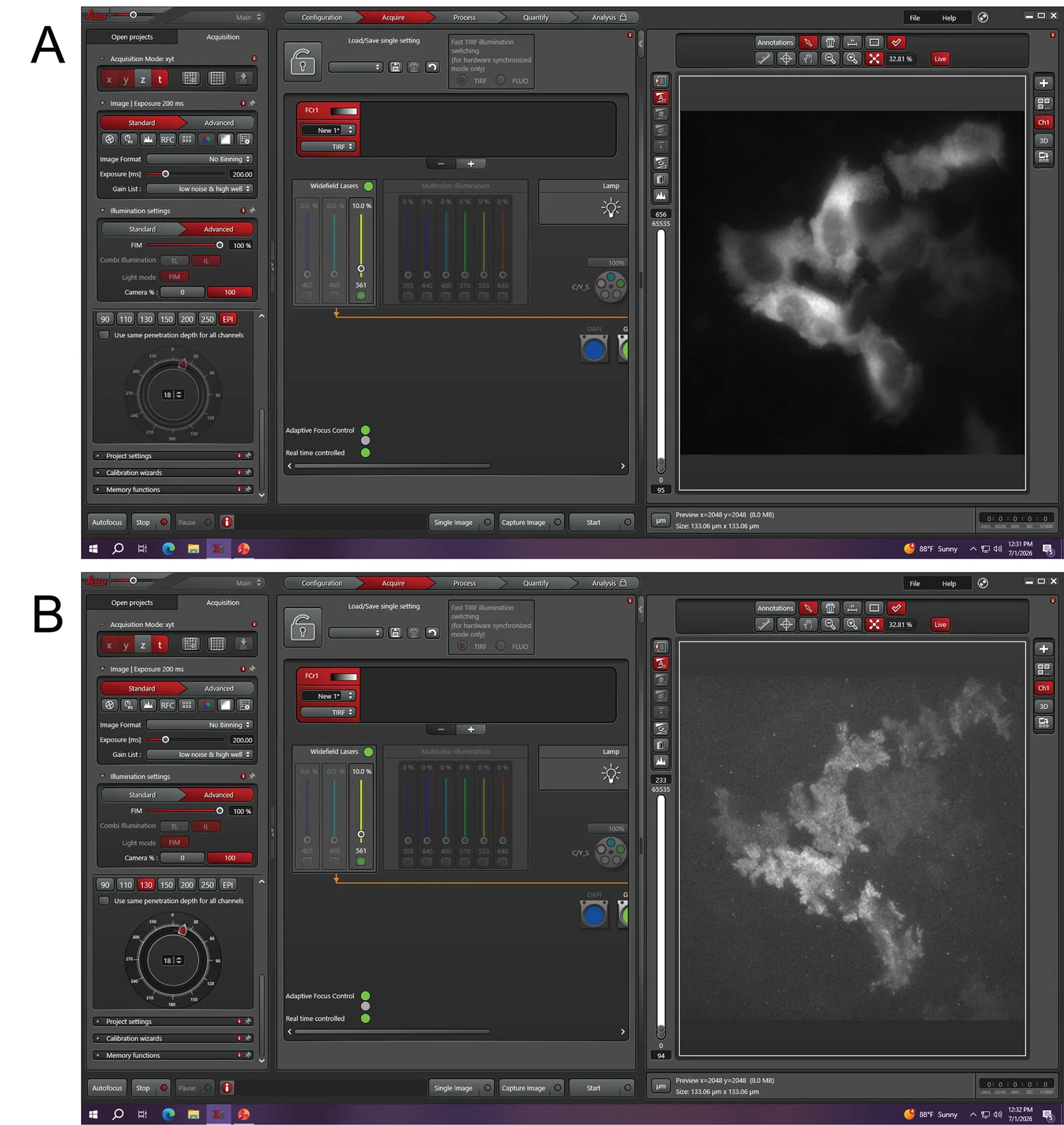
Comparison of TIRF and EPI modes. (A) Imaging in TIRF mode on the DSRed-T channel using the EPI setting. (B) Setting the TIRF angle and penetration depth prior to beginning the experiment. For additional background and details on distinguishing between TIRF and EPI imaging, please refer to [2].

9. Use the LasX software to add a second fluorescent channel. Set this channel to image GFP and turn the 480 nm LED to 50% power. Set the exposure time for this channel to 50 ms.

10. Set up the timed experiment by adding a time series in the *Acquisition* panel. Set the interval to 30 s and the number of frames acquired to 21. The total acquisition time for this experiment will be approximately 10 min.

11. The images will be collected in the order that the fluorescent channels are set up in the software. Make sure that the DSRed TIRF channel is first and the GFP channel is second. This gives one image of cells prior to blue light activation.

*Note: It is important to clear any prior tasks or focus map settings from the LASX Navigator window before beginning this single cell group experiment.*

12. Click on *Start* to begin the experiment. Recruitment to focal adhesions will be visible by the second or third frame in the timed sequence (**Figure 7**). In comparison to imaging using widefield mode, focal adhesion recruitment should be readily apparent and not require significant readjustment of the image to find the correct focal plane.

**Figure 7.**
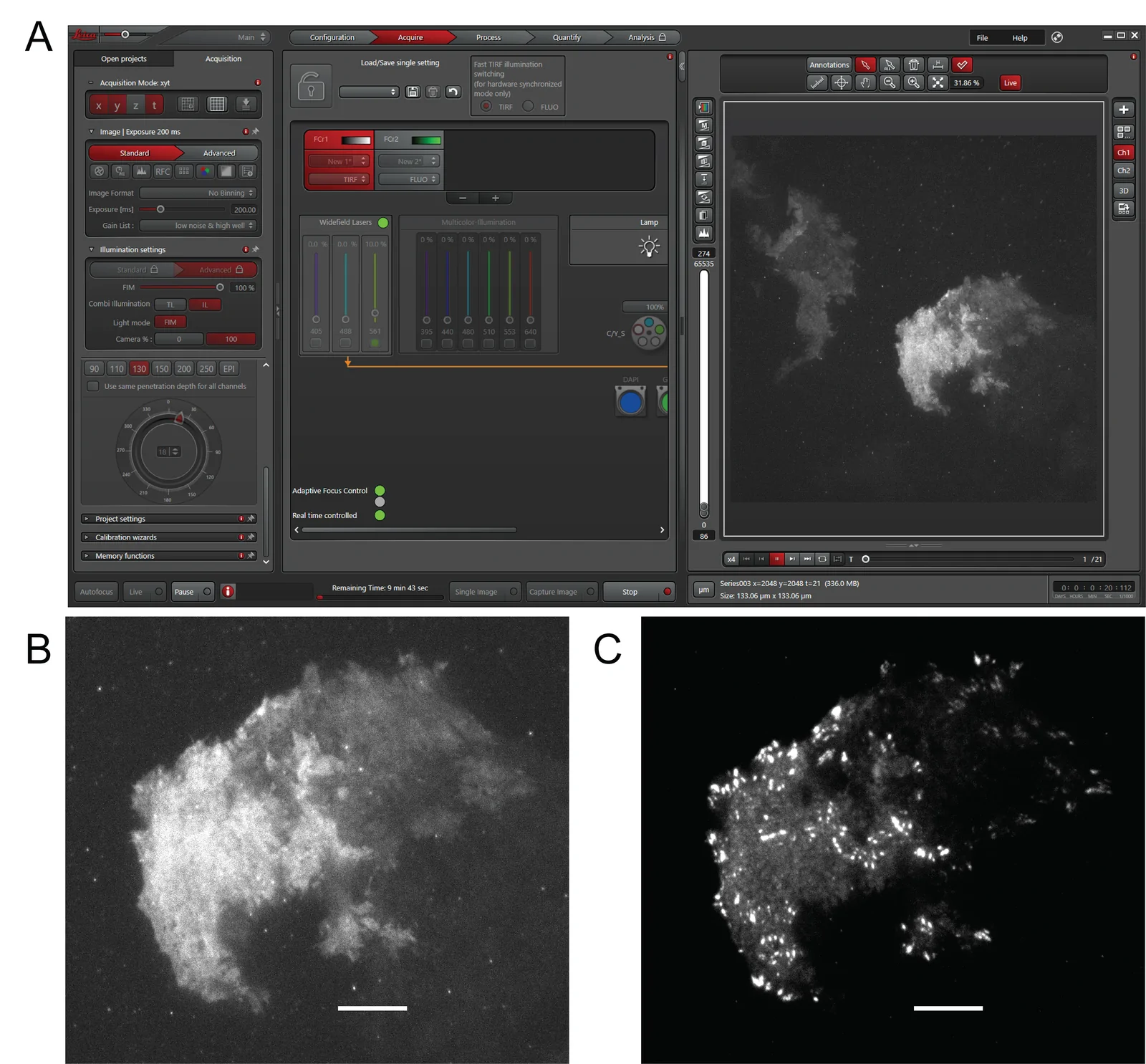
TIRF imaging of OptoProfilin. (A) The software is set to collect a TIRF image on the DSRed-T channel and a fluorescent image on the GFP channel. (B) TIRF imaging of cells pre-480-nm light activation. (C) TIRF image of cells post-480 nm light activation. TIRF mode greatly enhances the visibility of focal adhesions versus standard widefield fluorescence imaging. Scale bars = 10 μm.

13. After the 10-min acquisition sequence is complete, quickly remove the GFP acquisition channel from the software, and begin the acquisition again. This will enable imaging of OptoProfilin as it returns from the focal adhesions back to the cytosol.

*Note: Focal adhesions are more readily located in TIRF mode. This approach can be particularly useful in located focal adhesions in cell lines or under cell culture conditions in which focal adhesions are not abundant.*

**Patr III. Fixation and immunostaining of OptoProfilin cells**

**A. Preparation of HeLa cells for transfection.**

1. Grow HeLa cells in DMEM (10% FBS/1% Pen-Strep) at 37 °C and 5% humidity until they are 70%–80% confluent in a T75 growth flask with filter cap.

2. In a laminar flow hood, remove growth media and wash cells 1× with 5 mL of DPBS.

3. Add 5 mL of trypsin reagent to promote cell detachment and return cells to the incubator. Use a microscope to check cells for detachment from the flask every 5 min.

4. When cells are fully detached, return to the flow hood and add 5 mL of growth media. Use a 10 mL serological pipette to fully dissociate cells by pipetting up and down several times. Remove a 1 mL aliquot of cells from the flask and place them in a sterile tube. Count these cells using a hemacytometer.

5. Plate 200,000 cells per glass-bottom Mattek dish, then add 2 mL of growth media via pipette. Put the cover on the dish and distribute cells with a gentle rocking motion. Return to the incubator.

6. Allow cells to remain in the incubator overnight. The following day, check cells for confluency using a tissue culture microscope. Cells should be approximately 60%–70% confluent.

**Pause point:** If cells are not at the desired confluency, wait another 24 h before proceeding to the next step.

**B. Transfection preparation**

1. In a biological safety cabinet, aliquot 1,000 ng of OptoProfilin plasmid DNA into a sterile 1.5 mL tube.

2. Add 100 μL of serum-free DMEM to the DNA sample in the tube. Gently mix and spin down the solution with a benchtop centrifuge.

3. Add 3 μL of Calfectin transfection reagent to the DNA–DMEM solution. Gently mix by tapping the bottom of the tube and spin down the solution with a benchtop centrifuge.

**Critical:** Allow transfection solution to sit for at least 10 but no longer than 20 min, per manufacturer’s recommendation, as longer incubation times lead to reduced transfection efficiency and higher cell toxicity.

4. Add the transfection solution to the cells in the Mattek dish dropwise, using a circular motion to disperse the drops evenly over the dish, then return the cover to the dish and rock gently back and forth. Wrap the dish with aluminum foil to prevent unintended light activation and return it to the incubator. The experiment can begin after 16 h of incubation with the transfection complexes.

*Note: This step can be scaled as needed for the required number of dishes/replicates.*

**C. Illumination, fixation, and immunostaining of cells for confocal microscopy**

1. In a darkened room equipped with a red safe light, wash cells with DPBS and either restore 2 mL of prewarmed DPBS to the dish or treat the cells with 2 mL of cellular stress media. Return the dishes to the cell culture incubator for 10 min.

2. Bring the cells out of the incubator and illuminate them for 2 min with a blue LED light source (Sunlite LED Par30 Reflector, Item #80021, 4 W, 120 V) placed 10 cm from the cell culture dishes for 2 min. After illuminating, work quickly to remove the cellular stress media either by pipette or aspirator, wash gently and quickly with 1 mL of DPBS, then apply prewarmed fixative (4% paraformaldehyde) for 10 min.

3. Carefully remove the fixative solution and place it in the waste. Wash cells with DPBS (3 × 5 min) to remove excess fixative.

4. Permeabilize and block cells with antibody dilution buffer for 30 min at room temperature. Only add enough antibody dilution buffer to cover the cells in the glass-bottom portion of the dish (500 μL will be more than adequate).

5. Remove the blocking and permeabilization buffer by aspirator or pipette, then add the primary antibody (in this case, CST anti-Paxillin, 1:500 dilution in antibody dilution buffer). Return cover to dish, seal edges with parafilm, and incubate overnight at 4 °C.

6. The following day, remove primary antibody solution by pipette (this can be reused if desired) and wash cells 3 × 5 min with DPBS.

7. Incubate cells with secondary antibody (Alexa 488 conjugated goat anti-rabbit secondary; 1:1,000 in antibody dilution buffer), only using enough to cover the cells on the glass-bottom portion of the dish. Incubate cells for 1 h at room temperature, covered with foil to minimize light exposure.

8. Remove secondary antibody and wash cells 3 × 5 min with DPBS.

9. Add fresh DPBS and secure dish covers to dish bottoms with parafilm.

10. Image the resulting fixed cells with a confocal fluorescent microscope to confirm colocalization with the protein of interest.

**Part IV. Image processing for widefield and confocal microscopy**

**A. Processing of cells treated with stress solutions**

Imaging will be completed in FIJI/ImageJ [3]. For live cells treated with cellular stress solutions, stress-associated clusters can be quantified using the following steps (**Figure 8**):

1. Using Fiji/ImageJ, open the .LIF image sequences generated from the Leica LASX software.

2. Go to *Color > Split Channels* to isolate the red fluorescent channel.

3. Set the image threshold so that particles are trackable by the software.

a. Go to *Image > Adjust > Threshold*.

b. Set Default to B&W.

c. Check the following options: *Dark background* and *Don’t reset range*.

d. Move the slider to one of the final images in the sequence.

e. Adjust *Threshold* so that clusters appear as white dots on a black background.

f. Select *Apply*.

g. A *Convert Stack to Binary* window will appear. *Method* should be set to *Default* and *Background* to *Dark*.

h. Check *Black background* and *Create new stack* options.

i. Uncheck *Calculate threshold for each image*.

j. Select *OK*.

4. Go to *Analyze > Analyze particles* and use the *Analyze Particles* feature with the following settings:

a. Restrict particle size to 20–200 pixels (pixel units checked) and restrict circularity to 0.20–1.00.

b. Select *Show Outlines* from the dropdown menu.

c. Select the following options: *Display results, Clear results, Summarize, Add to Manager*, and *Overlay*.

d. Select *OK* to initiate the analysis.

5. Check the quality of particle capture with the software by visual inspection. If particles are not well defined in the acquired image, this automated analysis may be difficult, and manual particle counting will be necessary. Adjustment of particle size and circularity parameters may also be necessary.

6. Particle counts per frame, average areas, etc. will be found in the *Summary* window. This data can be copied and pasted into a spreadsheet for further analysis.

7. Report particle counts as the average number of particles/cell or as discrete numbers of particles/cell. Repeat this measurement for at least three replicate groups of 5–10 cells. Using particle counts from the beginning and end of each time course, plot these data in GraphPad Prism and determine significance with the appropriate statistical measure [1].

**B. Processing of immunostained cells**

For fixed cells subjected to immunostaining, use the following steps to assess the localization of OptoProfilin with the targets of antibody staining:

1. Using Fiji/ImageJ, open the Zeis format image files (.CZI).

2. Go to *Image > Color > Split Channels* to separate the fluorescent channels.

3. Manually adjust brightness and contrast (*Image > Adjust > Brightness/Contrast*).

4. Use the *Channels* tool (*Image > Color > Merge Channels*) to create an overlay image of the channels.

5. Using the non-merged channels, use the line tool to draw a line through the areas of apparent fluorescent localization of one of the images.

6. Click on the next image and select *Edit > Selection > Restore Selection* to make an identical line on that image. Do not click inside the image or the line will be lost.

7. Plot the fluorescence intensities of the selected areas by going to *Image > Stacks > Plot Z-axis Profile*. These results will appear in the results window and can be copied and pasted into a spreadsheet. Do this for both channels.

8. Plot both channels to visualize the extent of fluorescence overlay. The Y-axis will show fluorescence intensity, and the x-axis will show distance in microns. For light-activated OptoProfilin cells, the overlay of the mCherry and the Paxillin antibody immunofluorescence should be readily apparent. Further quantification can be performed if desired using Pearson’s Coefficient calculator in ImageJ (*Analyze > Colocalization > Coloc 2*) [3].

**Figure 8.**
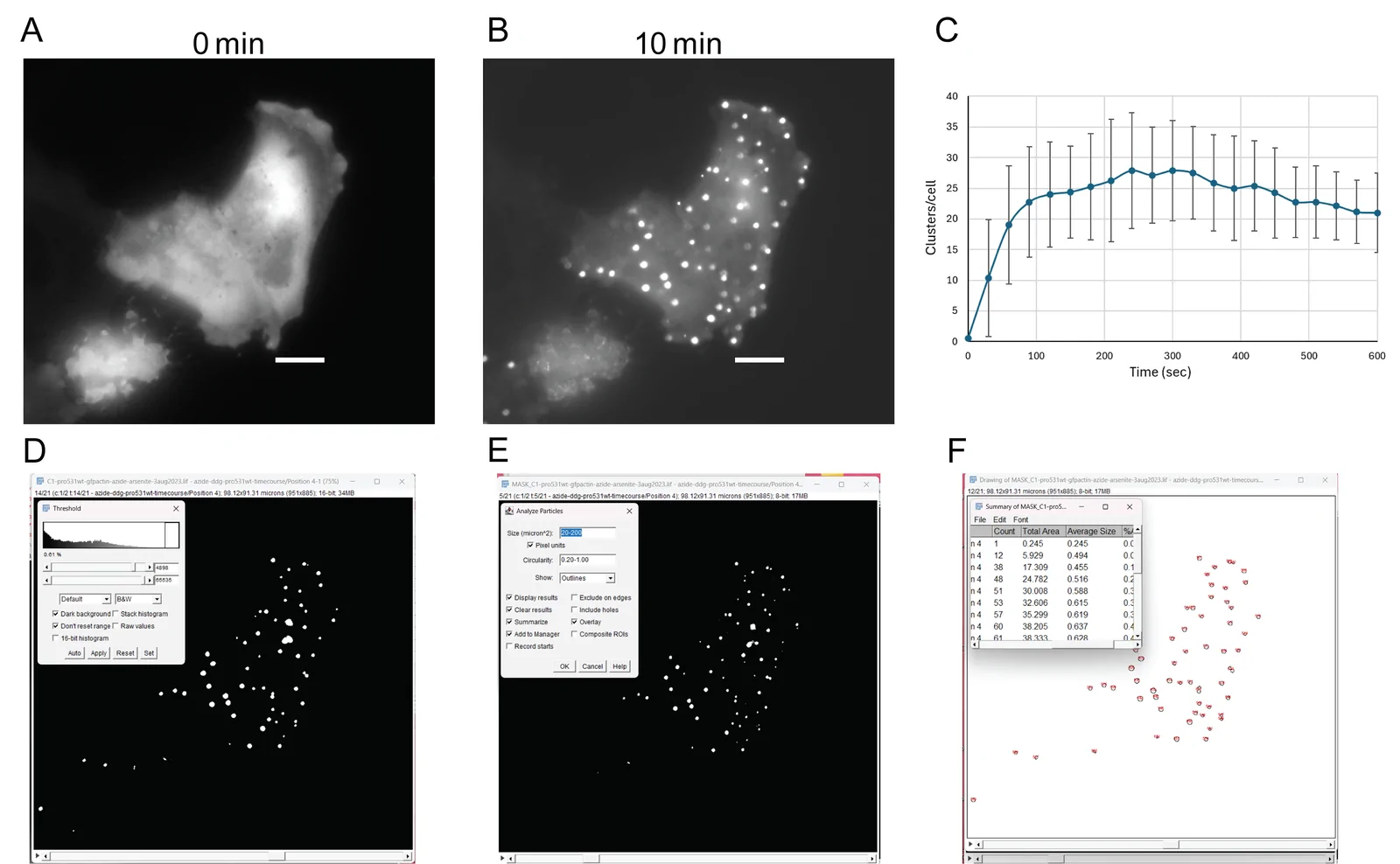
Analysis of experimental results in Fiji/ImageJ. (A) Cell prior to 480-nm light activation and (B) 10 min post-480 nm light activation. (C) Quantification of clusters/per cell versus time from n = 5 cell groups (error bars represent standard deviation). Clusters are counted in ImageJ by (D) setting a threshold that displays clusters only, (E) setting acquisition parameters in the *Analyze Particles* tool, and (F) evaluating the resulting particle counts. To determine the average number of clusters per cell, particle counts are taken from the time point of interest in the imaging sequence and divided by the number of OptoProfilin-expressing cells in the field of view. An example of this analysis is shown in panel C. Scale bars = 10 μm.

## Data analysis

Image analysis is performed using FIJI/ImageJ. Pearson correlation coefficients are calculated using the Coloc2 plugin. At least five independent biological replicates are recommended for each experimental condition. Cell populations should be scored manually or using automated segmentation workflows. Particle (i.e., cluster) counts should be assessed as either average number of particles per cell [total number of assessed particles in a single field of view/total number of cluster forming cells; often graphed as particle counts (y axis) vs. time (x axis)] or %cells displaying clusters (percentage of cells in a single field of view forming clusters after a defined activation interval). Differences in phenotypes (focal adhesion vs. stress-associated clusters) can be quantified by measuring lengths before and after stress application, as shown in Figure 5C. Statistical analyses may be performed using GraphPad Prism. Differences between groups can be assessed using Student’s t-test or ANOVA with appropriate post hoc corrections.

## Validation of protocol

This protocol has been validated by our laboratory in multiple mammalian cell lines, including HeLa, HEK293T, NIH 3T3, and Neuro-2a cells [1]. In HeLa cells, OptoProfilin localizes to focal adhesions under basal conditions and transitions to punctate VASP-containing condensates following energetic, oxidative, osmotic, or senescence-associated stress. Immunofluorescence confirms colocalization with Paxillin (focal adhesion marker) and VASP. The protocol was further validated using Profilin S138A and S138E mutants [1], demonstrating dependence on interaction with VASP for focal adhesion recruitment and stress-induced clustering [4].

## General notes and troubleshooting

**General notes**

1. HeLa cells provide the most robust focal adhesion phenotype of those tested so far.

2. Other cell types investigated did not have a robust focal adhesion phenotype, but OptoProfilin still responded to applied stress in these cell lines.

3. Excessive illumination can induce phototoxicity and should be minimized.

4. Expression levels should be optimized to avoid aggregation artifacts.

5. Prior to beginning recruitment experiments, all manipulations are done using the DSRed-T channel to avoid preliminary activation of OptoProfilin.

**Troubleshooting**

**Problem 1:** No OptoProfilin recruitment observed.

Possible cause: Low expression level.

Solution: Optimize transfection conditions and verify expression by fluorescence microscopy.

**Problem 2:** High background clustering.

Possible cause: Overexpression of OptoProfilin.

Solution: Reduce DNA quantity used for transfection.

**Problem 3:** Weak stress response.

Possible cause: Ineffective stress treatment.

Solution: Prepare fresh stress-inducing reagents and verify treatment times.
